# Rapid and transient oxygen consumption increase following acute HDAC/KDAC inhibition in Drosophila tissue

**DOI:** 10.1038/s41598-018-22674-2

**Published:** 2018-03-08

**Authors:** Lore Becker, Melanie Schmitt Nogueira, Caroline Klima, Martin Hrabe de Angelis, Shahaf Peleg

**Affiliations:** 1 German Mouse Clinic, Helmholtz Zentrum Munich, German Research Center for Environment and Health (GmbH), 85764 Neuherberg, Germany; 2 Munich Center of Integrated Protein Science and Biomedical Center, Ludwig-Maximilians University of Munich, Planegg-Martinsried, 80336 Germany; 3 German Center for Diabetes Research (DZD), Ingolstädter Landstr. 1, 85764 Neuherberg, Germany; 4 Chair of Experimental Genetics, School of Life Science Weihenstephan, Technische Universität München, Alte Akademie 8, 85354 Freising, Germany; 5 Laboratory for metabolism and epigenetics in brain ageing, Institute of Neuroregeneration & Neurorehabilitation, Qingdao University School of Medicine, 308 Ningxia Street, Qingdao, 266071 China

## Abstract

Epigenetic deregulation, such as the reduction of histone acetylation levels, is thought to be causally linked to various maladies associated with aging. Consequently, histone deacetylase inhibitors are suggested to serve as epigenetic therapy by increasing histone acetylation. However, previous work suggests that many non-histone proteins, including metabolic enzymes, are also acetylated and that post transitional modifications may impact their activity. Furthermore, deacetylase inhibitors were recently shown to impact the acetylation of a variety of proteins. By utilizing a novel technique to measure oxygen consumption rate from whole living tissue, we demonstrate that treatment of whole living fly heads by the HDAC/KDAC inhibitors sodium butyrate and Trichostatin A, induces a rapid and transient increase of oxygen consumption rate. In addition, our study indicates that the rate increase is markedly attenuated in midlife fly head tissue. Overall, our data suggest that HDAC/KDAC inhibitors may induce enhanced mitochondrial activity in a rapid manner. This observed metabolic boost provides further, but novel evidence, that treating various maladies with deacetylase inhibitors may be beneficial.

## Introduction

Epigenetic deregulation is associated with the onset and progression of many diseases^1–3^. For example, changes in lysine acetylation in histones, an important modification that alters chromatin structure and affects transcription activation, have been causally related with cancer, neurodegeneration, psychiatric disorders, various other maladies, and aging^2,4–9^. In many of these diseases, including cancer^4^ and cognitive decline^6,10,11^, lower histone acetylation and transcription deregulation are proposed as causal mechanisms; however, during early phases of aging, higher histone acetylation are observed^8,12^. As such, much effort has been aimed towards finding epigenetic treatments that increase histone acetylation levels.

Histone deacetylation is mediated by nuclear histone deacetylases (HDACs)^13^. Notably, many molecules that inhibit the activity of HDAC’s have been examined^14,15^. Among them are broad-spectrum classical HDAC inhibitors like Sodium Butyrate (SB), Trichostatin A (TSA), Veronistat (SAHA), and others. Treatment with these HDAC inhibitors increased histone acetylation and had beneficial impacts on cancer and neurodegeneration treatments, improved cognitive function, and others^5,10,11,14–20^.

Recent technological improvements in mass spectrometry analysis have revealed the presence of lysine acetylation in hundreds of non-histone proteins^13,21–24^. Many of these acetylated sites are located in mitochondria and can be deacetylated by class III deacetylases, the sirtuins, which are not sensitive to classical HDAC inhibitors such as SB, TSA and SAHA^13,25^. Nonetheless, numerous acetylated proteins, including transcription factors and metabolic enzymes involved in glycolysis and acetyl-CoA metabolism, are located in the cytoplasm and nucleus. Previously, it was shown that various HDACs, located in the cytoplasm and the nucleus, mediate the acetylation of various proteins^13^. As such, they should be referred to as lysine (K) deacetylases or KDACs. Importantly, acetylation of these non-mitochondrial metabolic enzymes impacts their activity^8,22,26,27^. KDAC inhibitors, such as SB and TSA, that can target KDACs in the cytoplasm could potentially increase the acetylation of metabolic enzymes and ultimately affect metabolic rates^25,28,29^.

It was previously shown in Drosophila that chronic reduction of KDAC1 (Rpd3) by RNAi treatment results in increased citrate synthase activity, a marker for mitochondrial activity^30^. In addition, chronic treatment with SB caused increased oxygen consumption in mice^31^. However, the relative effect of chronic KDAC inhibition on the acetylation of metabolic enzymes in contrast to complex transcriptional changes, mediated by altered histone acetylation that affects the abundance of metabolic enzymes, remains to be elucidated. Importantly, it is unclear whether acute and rapid KDACi treatment, which may not involve transcription, impacts metabolic activity.

We recently demonstrated that administration of SB and TSA to a whole Drosophila head caused increased oxygen consumption rate (OCR) after five cycles (Approximately half an hour) of measurement^28^. To gain further insight into the dynamic impact of acute KDAC inhibition on metabolism, we focused on characterizing the time-depended OCR changes that occur following KDAC inhibition in young and midlife male fly heads.

## Results

### Opposing trends in oxygen consumption rate in isolated mitochondria and whole head tissue

**Figure 1:**
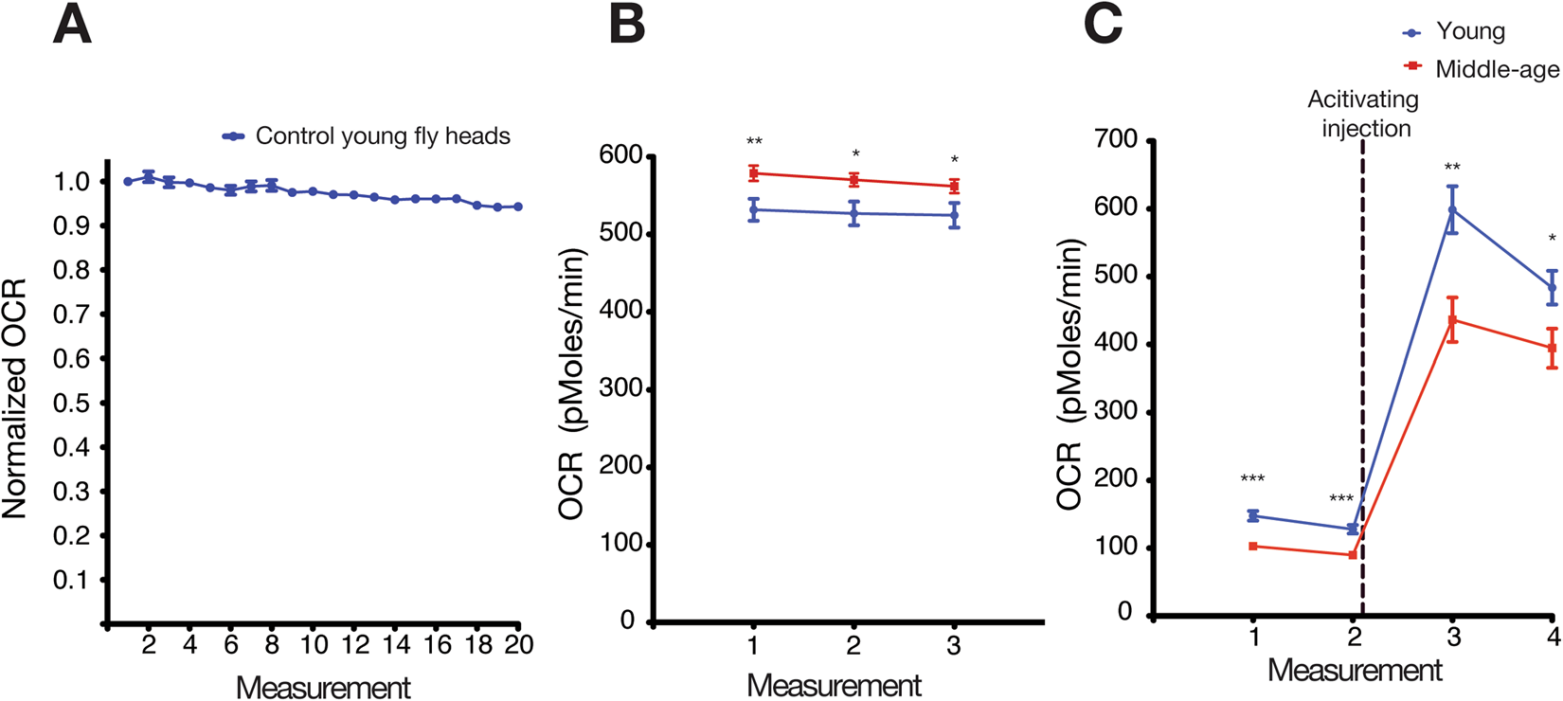
A novel method to measure dynamic oxygen consumption rate of whole living fly head tissue. (**A**) Young male fly head tissue display a stable **o**xygen consumption rate (OCR) over 20 consecutive measurements. (**B**) Three consecutive measurements of OCR in whole fly tissue show an increased OCR in midlife whole heads compared to young whole head. N = 20 young and 22 midlife. (**C**) Isolated mitochondria from midlife fly heads indicate reduced OCR compared to isolated mitochondria from young fly heads. N = 12 per group. (*P < 0.05, **P < 0.01, ***P < 0.001). Error bars indicate the S.E.M. in all the graphs.

Measuring oxygen consumption from isolated mitochondria is a common readout for cellular metabolic activity^32^. However, recent studies suggest that isolated mitochondria lack the complexity of whole cell tissue^12,33–35^. To address this problem, we implemented a novel technique to measure oxygen consumption rate from whole fly head (see methods). This technique enables the steady measurement of OCR in living male fly heads for at least 20 measurements (Fig. 1A and Supplementary Table 1).

We next compared the OCR of one week (young) and four week (midlife) old male fly heads^28^. Using a single measurement, we previously showed that OCR in midlife fly head was increased compared to the young group^28^. Interestingly, analysis of three consecutive measurements from whole tissue exhibited increased OCR in midlife tissue (Fig. 1B), but isolated mitochondria from midlife fly head, which lack cellular/tissue complexity^33,35^, exhibited decreased levels of OCR (Fig. 1C). While the reason for these conflicting results remain unclear and discussed elsewhere^12^, it is possible that older mitochondria are more fragile and thus break down more easily during mitochondrial isolation^34,35^. This emphasizes the importance of measuring OCR from whole tissue, which more closely reflects physiological complexity.

### Sodium butyrate induced a rapid and transient OCR increase in young fly heads

**Figure 2:**
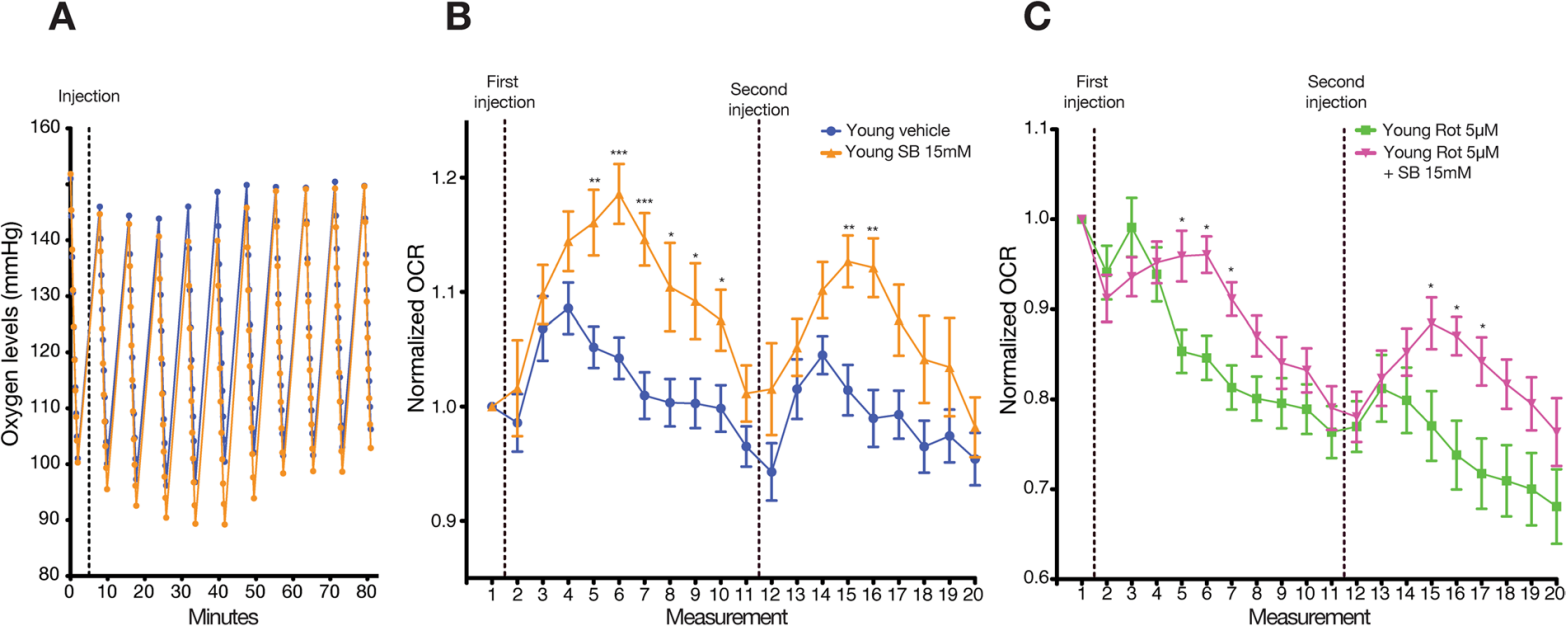
Sodium butyrate induces a dynamic and rapid increase in oxygen consumption rate in young fly whole head tissue. (**A**) Representative oxygen level changes showing decreased oxygen levels due to oxygen head consumption in control and SB treated samples. In SB treated fly heads, the oxygen drops to low levels and the OCR decreases at the last ‘ticks’. Therefore, we use the FIXED algorithm (See Methods). Each measurement (two minutes) consists of 10 sub-measurements referred to as a ‘tick’. The dashed line indicates the addition of SB. (**B**) Quantification of (**A**). Sodium butyrate (SB) induces a rapid and transient increase in oxygen consumption in the heads of young flies. A second injection after 10 measurements following the first injection results in a lower and shorter increase of OCR. Data was normalized to the measurement prior to addition of SB. The dashed lines indicate the addition of SB. N = 9 vehicle and 8 SB. (**C**) The addition of rotenone, a complex I inhibitor, significantly reduced OCR in whole fly head tissue. However, the addition of sodium butyrate induces increased OCR in the rotenone treated samples. The dashed line indicates the addition of rotenone or rotenone + SB. N = 9 per group. (*P < 0.05, **P < 0.01, ***P < 0.001). Error bars indicate the S.E.M. in all the graphs.

To evaluate the impact of sodium butyrate (SB) on OCR over time, we treated whole living head tissue with SB. Our data indicate that SB induced a significant and rapid increase in OCR after 4 measurements (Supplementary Table 1) following the drug treatment compared to the vehicle group (Fig. 2A and B). Induction of OCR peaked at 5 measurements and subsided after a total of 10 measurements following the addition of the drug (Supplementary Table 1) in the SB-treated group. A second addition of SB induced a second but lesser and more transient increase in OCR in the young fly head tissue. Inhibition of complex I of the respiratory chain by rotenone caused a significant reduction of OCR in whole fly tissue (Fig. 2C). Nonetheless, the addition of SB to rotenone treated heads caused increased OCR, supporting the hypothesis that SB is likely to stimulate OCR increase via alternative targets (Fig. 2C).

### Sodium butyrate induced a modest OCR increased in midlife fly heads

**Figure 3:**
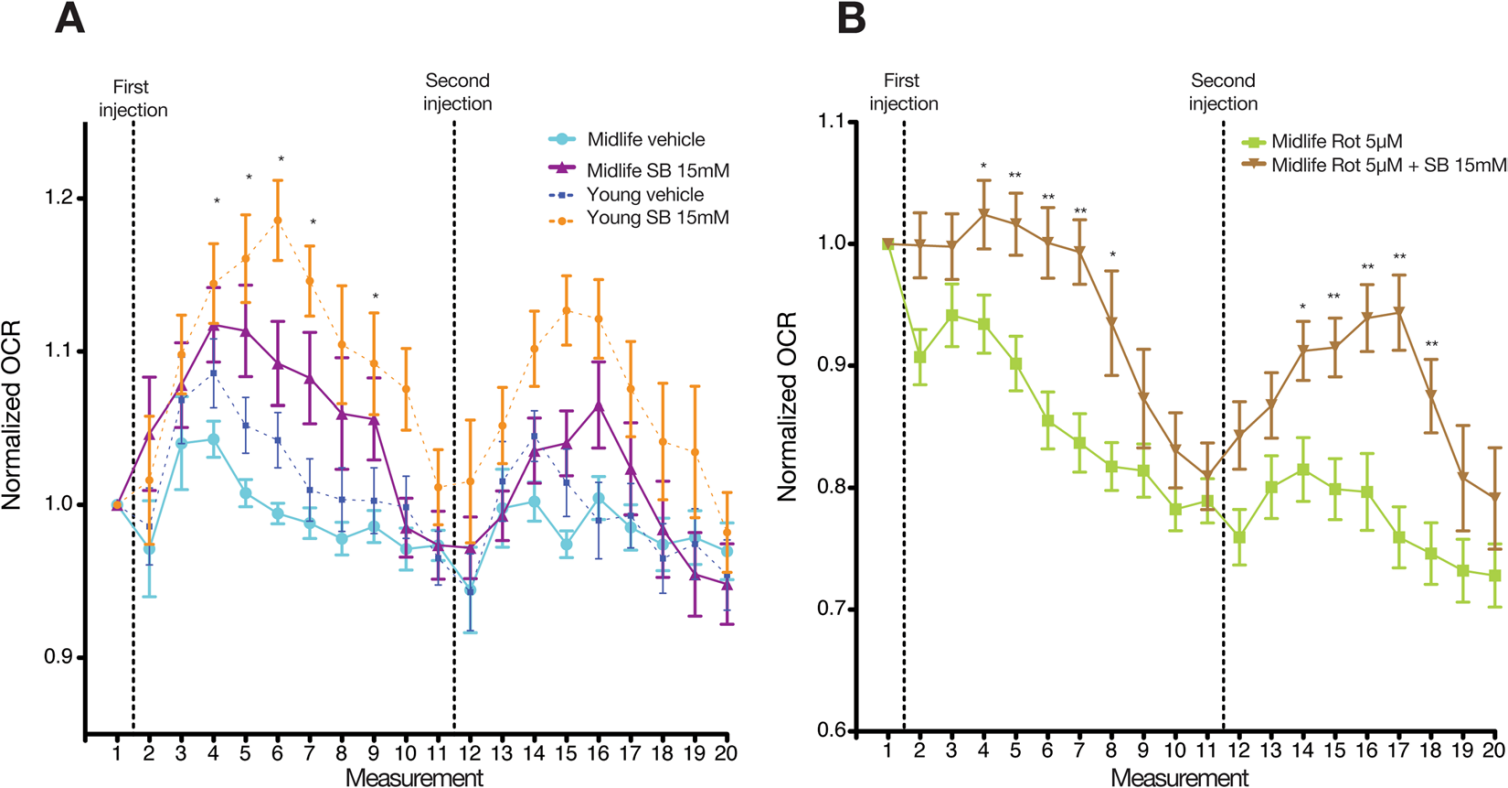
Sodium butyrate causes a lower and shorter increase in midlife fly whole head tissue. (**A**) Sodium butyrate (SB) induces a slight increase in oxygen consumption in the heads of midlife flies compared to the younger group. A second injection after 10 measurements following the first injection results in a lower and shorter increase of OCR. Data was normalized to the measurement prior to addition of SB. The dashed lines indicate the addition of SB. N = 9 per group. (**B**) The addition of rotenone significantly reduced the OCR in whole fly head tissue of midlife. Similarly to the results in Fig. 2C, the addition of sodium butyrate induces an increase in OCR in the rotenone treated samples. The dashed line indicates the addition of rotenone or rotenone + SB. N = 9 per group. (*P < 0.05, **P < 0.01) Error bars indicate the S.E.M. in all the graphs.

Sodium butyrate and other KDACi are currently promoted to treat various age-associated maladies^5,14,15,36^. While the beneficial impact of KDACi in treating age-related maladies is hypothesized to be mediated by histone acetylation and transcriptional changes, we recently demonstrated that SB causes a very rapid increase in oxygen consumption in midlife fly head after five cycles (Approximately half an hour) of measurement^28^. Similarly to young fly heads, SB induces a rapid increase of OCR as early as after 3 measurements following the addition of the drug (Fig. 3A). In contrast, the amplitude of the OCR increase is reduced and shorter compared to the young group (Fig. 2A). Likewise, a second consecutive injection of SB results in a mild and short OCR increase (Fig. 3A). Similarly to the young group, inhibition of the respiratory chain by rotenone caused a significant reduction of OCR in midlife fly heads (Fig. 3B). The addition of SB to rotenone treated heads also increased OCR in the midlife group (Fig. 3B).

### TSA induced a rapid and transient increase in OCR in whole young fly heads

Although sodium butyrate is considered a HDAC/KDAC inhibitor, it is also a metabolite. Indeed, several cell types within the colon utilize butyrate as a metabolite for energy production^15,37,38^. In fact, previous studies indicated that the addition of butyrate causes increased oxygen consumption in colonic cell types^39,40^. While this phenomenon may be due to the cells’ proximity to butyrate produced by local colonic bacteria^15,37^, we tested a second KDAC inhibitor to corroborate our original hypothesis/strengthen our results.

**Figure 4:**
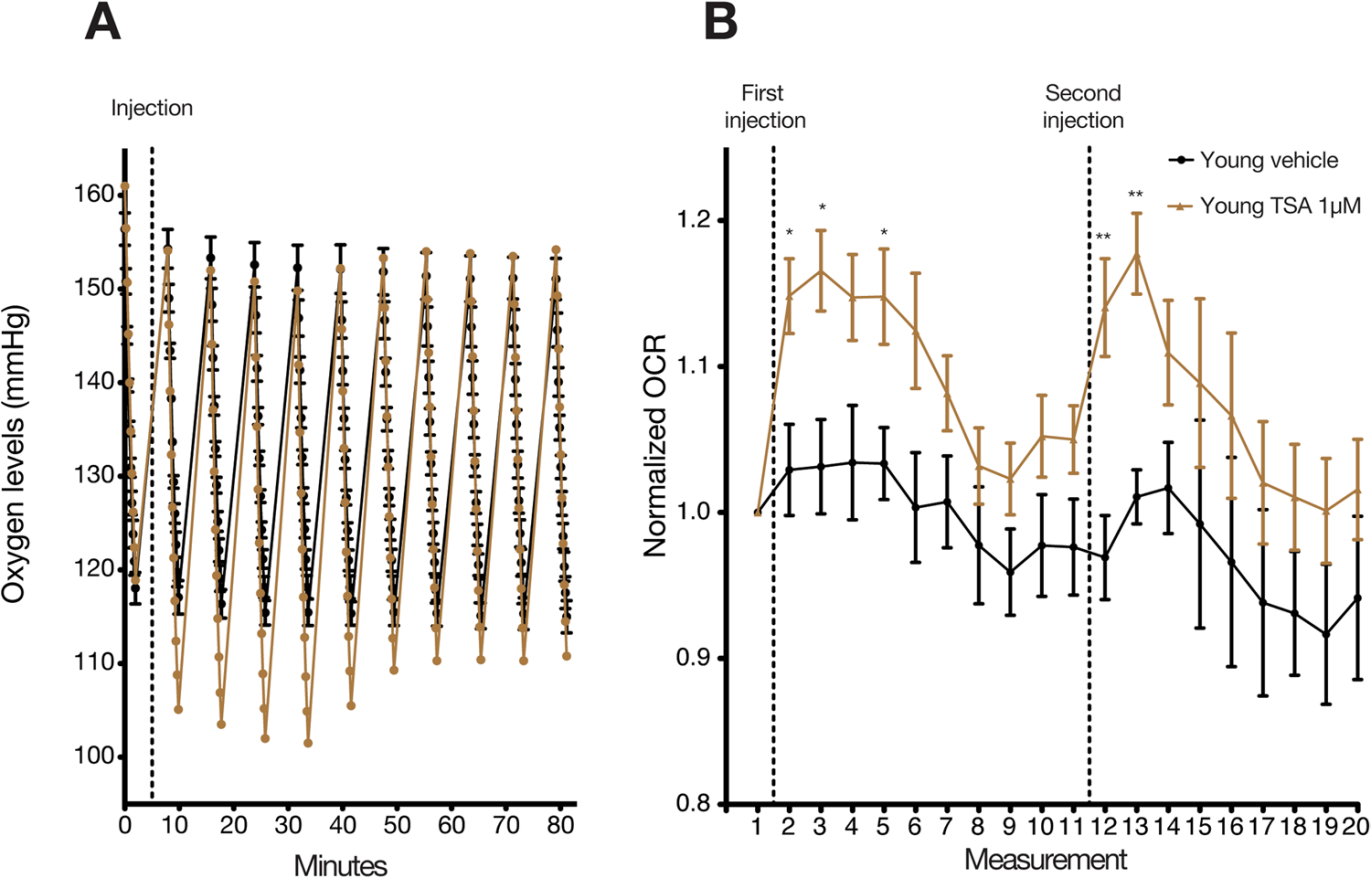
TSA causes a dynamic and very rapid increase in oxygen consumption rate in young fly whole head tissue. (**A**) Representative oxygen head consumption in control and TSA treated samples. Similarly to the results in Fig. 2A, in TSA treated fly heads, the oxygen drops to low levels and the OCR decreases at the last ‘ticks’. We use the FIXED algorithm to model the results (See Methods). The dashed line indicates the addition of TSA. (**B**) Quantification of (**A**). TSA induces a very rapid and transient increase in oxygen consumption in the heads of young flies. A second injection after 10 measurements following the first injection results in a shorter increase of OCR but not lower. Data was normalized to the measurement prior to addition of TSA. The dashed lines indicate the addition of TSA. N = 5 vehicle and 6 TSA. (*P < 0.05, **P < 0.01). Error bars indicate the S.E.M. in all the graphs.

We previously demonstrated that similarly to SB, TSA induces OCR increase after five cycles (Approximately half an hour) of measurement in whole young fly head^28^. Interestingly, compared to SB, TSA induced a faster increase in OCR, evident already in the first measurement following the addition of the drug (Fig. 4A,B and Supplementary Table 1). Of note, while TSA induced a more rapid response, the overall increase in OCR was less compared to SB treatment. Furthermore, a second injection of TSA after 70 minutes resulted in a second but more transient increase in OCR (Fig. 4B).

## Discussion

In this study, we used a novel approach to measure oxygen consumption of whole living tissue. The clear advantage is that this technique allows our determination in near-physiologic conditions, unlike classically isolated mitochondrial measurements^33^. For instance, in whole tissue, mitochondria cross talk with many other cellular processes that may influence mitochondrial activity. Furthermore, in a tissue cell-to cell communications still exist within a new tissue, a factor that may further influence metabolic rates^12^. Also, during mitochondria isolation, many posttranslational modifications (PTM’s) may be lost, resulting in metabolic change artifacts^12^; it is less likely that PTM’s are lost if the examined tissue is still alive.

This is important as our results suggest that PTM’s such as acetylation are of significant importance^28^. SB, and in particular TSA, induce rapid increases in OCR. Such quick OCR increase is likely to precede and be independent of new gene transcription following protein translation^41^, and therefore not directly mediated by histone acetylation. Indeed, our data suggests that SB and TSA impact OCR via non-histone protein acetylation, a dynamic process that can occur within the time-frame we observe^23,28^, but happens too quickly for results due to transcriptional changes^41^. The hypothesis that KDAC inhibitors impact metabolic activity independently from altering histone acetylation is valuable. It suggests that rather than epigenetic–mediated therapy, KDACi causes a metabolic “boost” that is crucial for KDACi-characterized benefits. For example, KDACi may cause an increase in mitochondrial activity in age-related disorders such as neurodegeneration characterized by reduced metabolic activity^36,42^. Furthermore, in cancer cells that exhibit altered metabolic activity, KDACi–induced metabolic changes could therapeutically target harmful cancer cells in a specific manner^5,14^. Further work is required to elucidate whether KDACi–induced metabolic increase is beneficial for various conditions affected by altered metabolic activity.

How do KDACi induce rapidly increased OCR? One possibility is that KDAC inhibition results from increased acetylation of cytosolic enzymes belonging to the glycolytic process^26^. If the result of this increased acetylation is increased glycolysis and mitochondrial pyruvate/acetyl-CoA levels and hence increase mitochondrial protein acetylation, it may cause a downstream increase of mitochondrial activity. Also, if the KDACi cause altered activity of enzymes, which shift the ADP/ATP balance or other relevant metabolites and cofactors, that may cause a change in the rate of oxidative phosphorylation.

It is important to note that while the activity of KDACs, with the exception of sirtuins, is believed to primarily occur in the nucleus and cytoplasm^13^, previous work had shown that several KDACs are present in the mitochondria during Xenopus development^43^. Furthermore, previous results in prostate cancer epithelial cells showed that KDAC7 is localized to the mitochondria^44^. Therefore, we cannot rule out the possibility that KDACi can inhibit KDACs that directly impact the acetylation of mitochondrial proteins. More work is needed to identify KDACi targets that result in increased OCR.

KDACi treatment has less impact on midlife fly head tissue. Since the OCR is already elevated in midlife, it is possible that the metabolic range is resistant to further increases^12^. We speculate that higher levels of protein acetylation in midlife limit further increase by KDACi^28^. Reaching this threshold level of OCR may explain why a second injection of KDACi to head tissue produces a weak OCR increase. Alternatively, it is possible the KDACi elicit a negative feedback that results in inhibition of OCR increase upon the second injection.

Large amounts of data suggest that epigenetic deregulation is linked with diseases and age-associated disorders, and manipulating histone acetylation can serve as KDACi-mediated therapy^2,8,9^. In fact, the combined KDACi impact on metabolism and epigenetic regulation may be crucial for successful treatment^8,16^. While KDAC inhibitors can cause increased levels of histone acetylation directly by inhibiting histone deacetylases, alternative mechanisms should be considered. First, KDACi can alter the acetylation and consequent activity of transcription factors that remodel DNA–histone interactions^13,24,25^. Second, increasing data demonstrate a relationship between metabolic activity and epigenetics^28,45–50^. For example, acetyl-CoA metabolism that produces the precursor for acetylation reactions significantly regulate levels of histone acetylation^8,28,45^. Our results indicate that KDACi can impact metabolic activity and support the hypothesis that KDACi indirectly increases histone acetylation by increasing the metabolic activity, resulting in higher levels of acetyl-CoA.

In summary, our results suggest a novel ability of KDAC inhibitors to induce a rapid and transient OCR increase. Our data support the concept that alternative, non-epigenetic mechanisms can be impacted by KDACi mediated therapy. Indeed, while histone acetylation was considered the main mechanism through which therapeutic benefits occur for various maladies, we now propose that modulating metabolism, protein acetylation, and metabolic-epigenetic relationships underlie the mechanism of successful KDACi-mediated therapy.

## Materials and Methods

### Flies

Flies (Canton) of mixed population of males and females were housed in 12 hour dark/light cycles at 25 °C, in 60% humidity and with free access to food as recently described, with an addition of yeast powder^28^. The food was changed every 2–3 days.

### Oxygen consumption assay from isolated mitochondria

Isolated mitochondria were extracted from 16 heads of one week or four week old male flies according to the manufacturer’s protocol (Seahorse/Agilent)^32^ and their oxygen consumption rate measured by the XF-96 well plate (Seahorse Agilent). Activation of the respiratory chain was initiated by ADP addition to the medium.

### Oxygen consumption assay in whole tissue

Oxygen consumption rate from whole tissue was measured as previously described^28^. In brief, male flies were anesthetized on an ice cold metal platform and their heads were removed by forceps. 16 fly heads were placed on a net (Seahorse/Agilent), carefully centered, and then inserted into a well (16 heads/well). Each well was filled with fresh Seahorse buffer (700 µl) containing freshly made 2.5% glucose. During preparation, the cart bridge was calibrated according to the manufacturer’s instruction and calibrated at 31–33 °C. Oxygen consumption measurements were collected in cycles of three minutes mixing, two minutes delaying phase, and a two minute measurement at 31–33 °C (Supplementary Table 1). While each cycle is calculated a total of 7 minutes, an extra of approximately half a minute of delay between mixing -delaying phase – measuring is added (Supplementary Table 1). Each two-minute measurement was divided into 10 sub-measurements referred to as “ticks” (See Figs 2A and 4A).

For direct injections, 77 µL and 85 µL (first and second injections, respectively) of fresh assay buffer were injected. A stock solution of fresh sodium butyrate (Sigma) of 150 mM was dissolved in the assay buffer before injection and pH adjusted, to a final concentration of 15 mM. Rotenone (Sigma) was dissolved in DMSO and a stock solution of 50 µM was prepared in the assay buffer to a final concentration of 5 µM. TSA (Sigma) was dissolved in DMSO and a stock solution of 10 µM was prepared in the assay buffer to a final concentration of 1 µM.

Oxygen consumption rate (OCR) was calculated with AKOS algorithm as previously described^51^. However, the oxygen levels decreased too rapidly and became too low (Anoxia) in the SB and TSA treated samples, resulting in a considerable decrease in OCR in the final sub-measurements (ticks) due to lower oxygen concentration (See Figs 2A and 4A). Therefore, the OCR in SB/TSA experiments was calculated with”FIXED” algorithm (Seahorse website).

### Statistical analysis

All data were tested positive for normal distribution. Data in Fig. 1 were analyzed with unpaired two‐tailed Student’s *t*‐test. For time course effects of drug treatments, the unpaired two‐tailed Student’s *t*‐test was performed and resulting p-values were corrected for multiple comparisons.

## Electronic supplementary material

Supplementary table 1
