# Supplementary material for: Rapid and transient oxygen consumption increase following acute HDAC/KDAC inhibition in Drosophila tissue
Source: Sci Rep. 2018 Mar 8;8:4199. doi: 10.1038/s41598-018-22674-2 (PMC5843646; doi:10.1038/s41598-018-22674-2)
Supplement: Supplementary file 1 — Supplementary table 1 [file 41598_2018_22674_MOESM1_ESM.pdf]

Rapid and transient oxygen consumption increase following acute HDAC/KDAC inhibition in Drosophila tissue

AAuthors: Lore Becker[1], Melanie Schmitt Nogueira[2#,] Caroline Klima[2#], Martin Hrabe de Angelis[1,3,4] and Shahaf Peleg[2,5*]

Affiliations:

[1]German Mouse Clinic, Helmholtz Zentrum Munich, German Research Center for Environment and Health (GmbH), 85764 Neuherberg, Germany.

[2]Munich Center of Integrated Protein Science and Biomedical Center, Ludwig-Maximilians University of Munich, Planegg-Martinsried 80336, Germany.

[3]German Center for Diabetes Research (DZD), Ingolstädter Landstr. 1, 85764 Neuherberg, Germany

[4]Chair of Experimental Genetics, School of Life Science Weihenstephan, Technische Universität München, Alte Akademie 8, 85354, Freising, Germany

[5]Laboratory for metabolism and epigenetics in brain ageing, Institute of Neuroregeneration & Neurorehabilitation, Qingdao University School of Medicine, 308 Ningxia Street, Qingdao, 266071, China.

#These authors contributed equally

*Correspondence: shahafpeleg3@googlemail.com (S.P.)

Supplementary Table 1: Timeline for whole tissue OCR measurement using XF24 islet plates.

Event	Time from start (min)	Time from first injection (min)	Event	Time from start (min)	Time from first injection (min)
Start	00:00:00	N/A	Mixing	01:51:12	00:46:18
Loading Cartridge	00:00:38	N/A	Waiting	01:54:22	00:49:28
Waiting	00:01:03	N/A	Measuring (8)	01:56:31	00:51:37
Mixing Calibrant	00:21:10	N/A	Mixing	01:58:53	00:53:59
Waiting after mixing	00:22:18	N/A	Waiting	02:02:03	00:57:09
Calibrating Cartridge	00:24:23	N/A	Measuring (9)	02:04:13	00:59:19
Final Calibration Measure	00:25:36	N/A	Mixing	02:06:34	01:01:40
Mixing	00:29:07	N/A	Waiting	02:09:44	01:04:50
Waiting	00:31:14	N/A	Measuring (10)	02:11:54	01:07:00
Mixing	00:33:20	N/A	Mixing	02:14:16	01:09:22
Waiting	00:35:28	N/A	Waiting	02:17:25	01:12:31
Mixing	00:37:34	N/A	Measuring (11)	02:19:34	01:14:40
Waiting	00:39:42	N/A	**Injection (2nd)**	**02:21:55**	**01:17:01**
Mixing	00:41:52	N/A	Mixing	02:22:07	01:17:13
Waiting	00:45:02	N/A	Waiting	02:25:16	01:20:22
Measuring	00:47:11	N/A	Measuring (12)	02:27:25	01:22:31
Mixing	00:49:33	N/A	Mixing	02:29:47	01:24:53
Waiting	00:52:43	N/A	Waiting	02:32:57	01:28:03
Measuring	00:54:53	N/A	Measuring (13)	02:35:07	01:30:13
Mixing	00:57:14	N/A	Mixing	02:37:29	01:32:35
Waiting	01:00:24	N/A	Waiting	02:40:38	01:35:44
Measuring (1)	01:02:34	N/A	Measuring (14)	02:42:47	01:37:53
**Injection (1st)**	**01:04:54**	**00:00:00**	Mixing	02:45:09	01:40:15
Mixing	01:05:06	00:00:12	Waiting	02:48:19	01:43:25
Waiting	01:08:16	00:03:22	Measuring (15)	02:50:29	01:45:35
Measuring (2)	01:10:26	00:05:32	Mixing	02:52:51	01:47:57
Mixing	01:12:47	00:07:53	Waiting	02:56:00	01:51:06
Waiting	01:15:57	00:11:03	Measuring (16)	02:58:09	01:53:15
Measuring (3)	01:18:07	00:13:13	Mixing	03:00:31	01:55:37
Mixing	01:20:29	00:15:35	Waiting	03:03:41	01:58:47
Waiting	01:23:38	00:18:44	Measuring (17)	03:05:51	02:00:57
Measuring (4)	01:25:47	00:20:53	Mixing	03:08:13	02:03:19
Mixing	01:28:09	00:23:15	Waiting	03:11:22	02:06:28
Waiting	01:31:19	00:26:25	Measuring (18)	03:13:31	02:08:37
Measuring (5)	01:33:29	00:28:35	Mixing	03:15:53	02:10:59
Mixing	01:35:50	00:30:56	Waiting	03:19:03	02:14:09
Waiting	01:39:00	00:34:06	Measuring (19)	03:21:13	02:16:19
Measuring (6)	01:41:10	00:36:16	Mixing	03:23:35	02:18:41
Mixing	01:43:32	00:38:38	Waiting	03:26:45	02:21:51
Waiting	01:46:41	00:41:47	Measuring (20)	03:28:54	02:24:00
Measuring (7)	01:48:51	00:43:57	End	03:31:16	02:26:22
